# Supplementary material for: An novel cloud task scheduling framework using hierarchical deep reinforcement learning for cloud computing
Source: PLoS One. 2025 Aug 21;20(8):e0329669. doi: 10.1371/journal.pone.0329669 (PMC12370147; doi:10.1371/journal.pone.0329669)
Supplement: Attachment — Submitted filename: Response to Reviewers.docx [file pone.0329669.s002.docx]

**Reviewer #1**:

Comment 1: The paper proposes a novel cloud task scheduling framework using hierarchical deep reinforcement learning (DRL) to address the challenges of large-scale task scheduling in cloud computing. The framework defines a set of virtual machines (VMs) as a VM cluster and employs hierarchical scheduling to allocate tasks first to the cluster and then to individual VMs. The scheduler, designed using DRL, adapts to dynamic changes in the cloud environment by continuously learning and updating network parameters. Experimental results demonstrate that this approach effectively balances cost and performance, optimizing objectives such as load balance, cost, and overdue time. One potential shortcoming of the proposed hierarchical deep reinforcement learning (DRL) framework for cloud task scheduling could be its complexity and computational overhead. Implementing and maintaining a DRL-based scheduler requires significant computational resources and expertise in machine learning. Additionally, the continuous learning and updating of network parameters might introduce latency, which could impact real-time task scheduling efficiency. How this could be handled in the proposed methodology? Furthermore, the framework’s performance heavily depends on the quality and quantity of training data, which might be challenging to obtain and maintain in a dynamic cloud environment.

Answer: Thanks for good advice. The shortcomings has revised in ABSTRACT and Conclusions.

ABSTRACT: There are still shortcomings in the method used in this article. Firstly, the continuous learning and updating of network parameters might introduce latency, which could impact real-time task scheduling efficiency. Furthermore, the framework's performance heavily depends on the quality and quantity of training data, which might be challenging to obtain and maintain in a dynamic cloud environment.

Comment 2: Contributions could be highlighted in the introduction sections

Answer: Thanks for good advice. Contributions has been highlighted in the introduction sections

This research proposes an innovative Hierarchical Intelligent Task Scheduling framework (HITS) based on the hierarchical deep reinforcement learning algorithm to address the challenge of large-scale task scheduling in cloud computing. Compared with traditional methods, HITS exhibits significant advantages. Firstly, through hierarchical partitioning and effective manipulation of the solution space, it accelerates the task scheduling process and simultaneously optimizes the task overdue time and cost, which is particularly crucial in large-scale task scheduling scenarios. Secondly, the model structure and return function of deep reinforcement learning are meticulously designed in accordance with the dynamic characteristics of the cloud environment. In response to the dynamic variation in the number of virtual machines, by modeling the Gaussian distribution of relevant features and using it as state information, the model can adaptively adjust. For different load conditions, a unique reward function is designed, which feeds back rewards based on the load to drive the model to learn corresponding decision-making strategies, thereby achieving efficient and intelligent task scheduling in the complex and variable cloud environment.

Comment 3: The quality, symmetry and the dimensions of figures needs to be improved and enhanced for formal presentation.

Answer: Thanks for good advice. The figures throughout the text have been revised.

Comment 4: The methodology section is not distinguishing that how this particular technique is better than existing work.

Answer: Thanks for good advice. The following content has been added to Chapter Three of Materials and methods.

Advantages of the Hierarchical Deep Reinforcement Learning Technology Employed in This Research over Existing Works:

Advantages of the Hierarchical Architecture: Compared with traditional single-layer task scheduling methods, the hierarchical architecture of HITS can effectively reduce the complexity of the problem. By dividing the task scheduling process into two levels, name-ly the cluster level and the virtual machine level, the decision space at each level is dimin-ished, and the scheduling efficiency is enhanced. In cluster-level scheduling, clusters suitable for task processing can be rapidly screened out, avoiding a global search among all virtual machines, thereby significantly shortening the task allocation time. Meanwhile, this hierarchical approach is also conducive to resource management and optimization, better balancing the loads among different clusters and virtual machines and improving resource utilization.

Adaptability of the Deep Reinforcement Learning Model: The deep reinforcement learning model in this research, through a meticulously designed state space and return function, demonstrates remarkable adaptability to the dynamic changes of the cloud environment. Unlike traditional rule-based or heuristic scheduling algorithms, the deep reinforcement learning model can automatically learn and adapt to the dynamic changes of tasks and resources in the cloud environment. For instance, by modeling the Gaussian distribution of changes in the number of virtual machines, the model can promptly perceive the increase or decrease of virtual machine resources and adjust the task allocation strategy accordingly. When confronted with different load situations, the unique reward function can guide the model to make a reasonable trade-off between task overdue time and cost, thereby achieving satisfactory scheduling performance under various complex load conditions.

Learning and Optimization Capabilities: The deep reinforcement learning model possesses powerful learning and optimization capabilities. Compared with traditional static scheduling algorithms, it can continuously learn in the process of ongoing task scheduling and constantly optimize its own scheduling strategy. Through techniques such as experience replay and target network, the model can effectively utilize historical empirical data for learning, avoid getting trapped in local optimal solutions, and gradually converge to a more optimal scheduling strategy. Such learning and optimization capabilities enable the HITS framework to continuously adapt to the changes of the cloud environment and continuously improve the efficiency and quality of task scheduling during long-term operation.

Comment 5: The methodology section hardly cites any literature for the formulae and state-of-the-art techniques.
Answer: Thanks for good advice. Add the corresponding citations of Reinforcement Learning and Double Deep Q-Network (DDQN) and Playing Atari with Deep Reinforcement Learning. Add four formulae in the methodology section.

30. R. S. Sutton, A. G Barto. Reinforcement Learning: An Introduction. IEEE Transactions on Neural Networks, 1998, 9(5):1054.

31. V. Mnih, K. Kavukcuoglu, D. Silver, A. Graves, I. Antonoglou, D. Wierstra, & M. Riedmiller. Playing Atari with Deep Reinforcement Learning. Computer Science, 2013.

Comment 6: Paper needs to be revised for grammatical errors and typos.

Answer: Thanks for good advice. The grammatical errors and typos of paper has been revised by native English speakers.

Comment 7: Article needs to be proofread from the native English speaker or rewritten in the academic writing.

Answer: Thanks for good advice. The paper has been revised by native English speakers.

Comment 8: The abstract needs to showcase the numerical finding of the research study to reflect the contribution in the field.

Answer: Thanks for good advice. The abstract has been revised.

With the increasing popularity of cloud computing services, their large and dynamic load char-acteristics have rendered task scheduling an NP-complete problem. Aiming at the problem of large-scale task scheduling in cloud computing environment, the paper proposes a novel cloud task scheduling framework using hierarchical deep reinforcement learning (DRL) to address the challenges of large-scale task scheduling in cloud computing. The framework defines a set of virtual machines (VMs) as a VM cluster and employs hierarchical scheduling to allocate tasks first to the cluster and then to individual VMs. The scheduler, designed using DRL, adapts to dynamic changes in the cloud environments by continuously learning and updating network parameters. Experiments demonstrate that it skillfully balances cost and performance. In low-load situations, costs are reduced by using low-cost nodes within the Service Level Agreement (SLA) range; in high-load situations, resource utilization is improved through load balancing. Compared with classical heuristic algorithms, it effectively optimizes load balancing, cost, and overdue time, achieving a 10% overall improvement. Experimental results demonstrate that this approach effectively balances cost and performance, optimizing objectives such as load balance, cost, and overdue time. One potential shortcoming of the proposed hierarchical deep reinforcement learning (DRL) framework for cloud task scheduling could be its complexity and computational overhead. Implementing and maintaining a DRL-based scheduler requires significant computational resources and expertise in machine learning. There are still shortcomings in the method used in this article. Firstly, the continuous learning and updating of network parameters might introduce latency, which could impact real-time task scheduling efficiency. Furthermore, the framework's performance heavily depends on the quality and quantity of training data, which might be challenging to obtain and maintain in a dynamic cloud environment.

Comment 9: The introduction needs to be providing rationale of the study, and brief literature review of existing studies, which is hard to differentiate in its current form.

Answer: Thanks for good advice. The introduction has been revised.

Cloud computing is a resource delivery and usage model. Service providers integrate a large number of nodes into a unified resource pool through virtualization technology, and users obtain the required computing resources through the network [1]. Cloud computing, as one of the core infrastructures in the current field of information technology, faces increasing pressure in task scheduling with the rapid development of big data, the Internet of Things, and 5G technologies. Task scheduling is an important research direction in cloud computing, whose essence is to reasonably allocate user requests to computing nodes for processing. However, this generates a huge solution space, and the optimal solution can-not be obtained in polynomial time, so the task scheduling of cloud computing is an un-certain NP problem [2,3].

Traditional task scheduling methods, whether heuristic algorithms [4] based on simple rules or some metaheuristic algorithms [5], exhibit numerous limitations when dealing with large-scale and dynamically changing cloud tasks [6]. For instance, heuristic algorithms often lack adaptability to complex environmental changes and have difficulty in flexibly adjusting scheduling strategies under different load and resource conditions. Although metaheuristic algorithms can perform global optimization to a certain extent, they have complex parameter settings and high computational overhead, making them difficult to be effectively applied in cloud task scheduling scenarios with high real-time requirements.

Many researchers have studied this problem and proposed heuristic and metaheuristic algorithms to solve it. But the actual cloud computing environment is complicated and dynamic, and traditional methods cope poorly with it. Re-searchers are using reinforcement learning (RL) and deep reinforcement learning (DRL) for learning capabilities to solve the dynamic scheduling problem of cloud computing [7-9]. Due to the diversity of user requests and resources, different quality of service (QoS) constraints must be simultaneously met, and how to respond to large-scale user requests while meeting the requirements of cloud service providers is an urgent problem. Intelligent scheduling algorithms are essential to overcome the difficulties of large-scale task scheduling. In this research, a hierarchical intelligent task scheduling framework (HITS) based on a hierarchical DRL algorithm is proposed. In the scheduling framework, a col-lection of VMs is called a VM cluster. When the framework receives a task request, it allocates the task to a cluster, and then to a VM via the task scheduler inside the cluster. We apply DRL technology to the scheduler, and through the design of the state space and re-turn function of each layer, it can adapt to the dynamic changes of the cloud computing environment, and adjust its scheduling strategy through continuous learning.

Based on these current situations, we propose to adopt the hierarchical deep reinforcement learning technology to address the cloud task scheduling problem. Deep reinforcement learning has powerful learning capabilities and adaptability to complex environments. It can automatically optimize scheduling strategies through continuous interaction and learning with the cloud environment. The hierarchical architecture helps to decompose large-scale problems into manageable sub-problems, improving decision-making efficiency and the system's scalability. We expect that through this innovative method, it is possible to meet the cost control requirements of cloud service providers while providing users with more efficient and reliable services, achieving a comprehensive improvement in multiple aspects such as performance, cost, and flexibility in cloud computing task scheduling, filling the gaps of traditional methods in handling large-scale and dynamic cloud task scheduling, and promoting the further development and application of cloud computing technology in the modern information technology system.

Cost and load balancing are two crucial objectives in cloud task scheduling. From the perspective of cost, cloud service providers need to reduce the cost of resource usage as much as possible to enhance profit margins while meeting user requirements. Different types of virtual machines have diverse cost structures, including computing cost, storage cost, and bandwidth cost. Our scheduler, through the deep reinforcement learning algorithm, comprehensively considers the resource requirements of tasks and the cost characteristics of virtual machines during the task allocation process. For example, when a task arrives, the scheduler evaluates the idle resource situation and the corresponding cost of the virtual machines within each cluster and preferentially assigns the task to the combination of virtual machines or clusters that can meet the task requirements and have a lower cost. Such an approach can effectively reduce the overall cost of task execution and improve the cost-effectiveness of resources.

For load balancing, the motivation is to ensure that the utilization rates of various resource nodes (clusters and virtual machines) in the cloud environment are relatively balanced and avoid situations where some nodes are overloaded while others are idle. This not only helps to improve the overall performance and stability of the system but also extends the service life of hardware devices. In the decision-making process, our scheduler takes the load situations of clusters and virtual machines as important state information and inputs it into the deep reinforcement learning model. By designing a reasonable return function, positive rewards are given to scheduling decisions that can achieve load balancing, and vice versa. For example, when the standard deviation of the virtual machine loads within a cluster is small, indicating a relatively balanced load, the scheduler tends to continue assigning tasks to this cluster. When the load of a certain virtual machine is too high, the scheduler will consider assigning subsequent tasks to other virtual machines or clusters with lighter loads, thereby dynamically adjusting the task allocation strategy to achieve load balancing of resources in the cloud environment and reducing performance bottlenecks and resource waste caused by uneven loads.

**Response to Reviewer #2**

Comment 1: How the hierarchical deep reinforcement learning helps in scheduling of tasks in cloud paradigm?

Answer: Thanks for good advice. Please refer to section “3.4 Algorithm Design” for relevant content.

Hierarchical deep reinforcement learning plays a crucial role in task scheduling in the cloud model. In the cloud environment, the scale of tasks and resources is vast and highly complex and variable. The hierarchical architecture divides task scheduling into two levels: the cluster level and the virtual machine level. At the cluster level, through the deep reinforcement learning algorithm, the suitability of tasks and clusters is evaluated and decisions are made. It can quickly screen out clusters with appropriate resource combinations (such as computing power, bandwidth, etc.), avoiding a global search among all virtual machines, thereby significantly reducing the complexity of the decision space and improving the initial efficiency of task allocation. At the virtual machine level, deep reinforcement learning further makes refined scheduling decisions for the virtual machines within the cluster. Taking into account dynamic information such as the real-time load and task processing history of the virtual machines, it determines the specific virtual machine that is most suitable for executing the task. The combination of this hierarchical structure and deep reinforcement learning enables the scheduler to gradually optimize the scheduling strategy in a large-scale cloud environment according to the dynamic changes of tasks and resources, achieving efficient task allocation and resource utilization and effectively addressing the challenges of complexity and dynamism in task scheduling under the cloud model.

Comment 2: Abstract should be concise and accurate

Answer: Thanks for good advice. The abstract has been adjusted as follows.

With the increasing popularity of cloud computing services, their large and dynamic load char-acteristics have rendered task scheduling an NP-complete problem. Aiming at the problem of large-scale task scheduling in cloud computing environment, the paper proposes a novel cloud task scheduling framework using hierarchical deep reinforcement learning (DRL) to address the challenges of large-scale task scheduling in cloud computing. The framework defines a set of virtual machines (VMs) as a VM cluster and employs hierarchical scheduling to allocate tasks first to the cluster and then to individual VMs. The scheduler, designed using DRL, adapts to dynamic changes in the cloud environments by continuously learning and updating network parameters. Experiments demonstrate that it skillfully balances cost and performance. In low-load situations, costs are reduced by using low-cost nodes within the Service Level Agreement (SLA) range; in high-load situations, resource utilization is improved through load balancing. Compared with classical heuristic algorithms, it effectively optimizes load balancing, cost, and overdue time, achieving a 10% overall improvement. Experimental results demonstrate that this approach effectively balances cost and performance, optimizing objectives such as load balance, cost, and overdue time. One potential shortcoming of the proposed hierarchical deep reinforcement learning (DRL) framework for cloud task scheduling could be its complexity and computational overhead. Implementing and maintaining a DRL-based scheduler requires significant computational resources and expertise in machine learning. There are still shortcomings in the method used in this article. Firstly, the continuous learning and updating of network parameters might introduce latency, which could impact real-time task scheduling efficiency. Furthermore, the framework's performance heavily depends on the quality and quantity of training data, which might be challenging to obtain and maintain in a dynamic cloud environment.

Comment 3: What is the motivation behind cost, load balancing and how it will be handled by your scheduler and explain about its impact.
Answer: Thanks for good advice. Please refer to section “Introduction” for relevant content.

Cloud computing, as one of the core infrastructures in the current field of information technology, faces increasing pressure in task scheduling with the rapid development of big data, the Internet of Things, and 5G technologies. Traditional task scheduling methods, whether heuristic algorithms based on simple rules or some metaheuristic algorithms, exhibit numerous limitations when dealing with large-scale and dynamically changing cloud tasks. For instance, heuristic algorithms often lack adaptability to complex environmental changes and have difficulty in flexibly adjusting scheduling strategies under different load and resource conditions. Although metaheuristic algorithms can perform global optimization to a certain extent, they have complex parameter settings and high computational overhead, making them difficult to be effectively applied in cloud task scheduling scenarios with high real-time requirements.

Based on these current situations, we propose to adopt the hierarchical deep reinforcement learning technology to address the cloud task scheduling problem. Deep reinforcement learning has powerful learning capabilities and adaptability to complex environments. It can automatically optimize scheduling strategies through continuous interaction and learning with the cloud environment. The hierarchical architecture helps to decompose large-scale problems into manageable sub-problems, improving decision-making efficiency and the system's scalability. We expect that through this innovative method, it is possible to meet the cost control requirements of cloud service providers while providing users with more efficient and reliable services, achieving a comprehensive improvement in multiple aspects such as performance, cost, and flexibility in cloud computing task scheduling, filling the gaps of traditional methods in handling large-scale and dynamic cloud task scheduling, and promoting the further development and application of cloud computing technology in the modern information technology system.

Cost and load balancing are two crucial objectives in cloud task scheduling. From the perspective of cost, cloud service providers need to reduce the cost of resource usage as much as possible to enhance profit margins while meeting user requirements. Different types of virtual machines have diverse cost structures, including computing cost, storage cost, and bandwidth cost. Our scheduler, through the deep reinforcement learning algorithm, comprehensively considers the resource requirements of tasks and the cost characteristics of virtual machines during the task allocation process. For example, when a task arrives, the scheduler evaluates the idle resource situation and the corresponding cost of the virtual machines within each cluster and preferentially assigns the task to the combination of virtual machines or clusters that can meet the task requirements and have a lower cost. Such an approach can effectively reduce the overall cost of task execution and improve the cost-effectiveness of resources.

For load balancing, the motivation is to ensure that the utilization rates of various resource nodes (clusters and virtual machines) in the cloud environment are relatively balanced and avoid situations where some nodes are overloaded while others are idle. This not only helps to improve the overall performance and stability of the system but also extends the service life of hardware devices. In the decision-making process, our scheduler takes the load situations of clusters and virtual machines as important state information and inputs it into the deep reinforcement learning model. By designing a reasonable return function, positive rewards are given to scheduling decisions that can achieve load balancing, and vice versa. For example, when the standard deviation of the virtual machine loads within a cluster is small, indicating a relatively balanced load, the scheduler tends to continue assigning tasks to this cluster. When the load of a certain virtual machine is too high, the scheduler will consider assigning subsequent tasks to other virtual machines or clusters with lighter loads, thereby dynamically adjusting the task allocation strategy to achieve load balancing of resources in the cloud environment and reducing performance bottlenecks and resource waste caused by uneven loads.

Comment 4: There are no contributions written by the authors in the article.

Answer: Thanks for good advice. Please refer to section “Conclusions” for relevant content.

We propose a hierarchical framework for large-scale online task scheduling to reduce task cost and overdue time. The uniqueness of this framework lies in the meticulously constructed state space and return function. The state space effectively models the uncertainties of the cloud environment by integrating multi-dimensional information of tasks, clusters, and virtual machines (such as resource requirements, processing capabilities, cost, etc.), and applying statistical methods like averaging and standard deviation to handle the characteristics of the dynamic environment. The return function flexibly trades off and optimizes between task overdue time and cost according to the load dynamics of the cloud environment, enabling the scheduler to automatically learn and adopt the most appropriate scheduling strategy under different load conditions, significantly enhancing the intelligence level of task scheduling.

Verified by experiments, in comparison with a variety of traditional task scheduling algorithms (such as Random, Round - Robin, Min - Min, Dominant Resource Fairness, etc.), the HITS framework exhibits distinct advantages in multiple key performance indicators. In terms of load balancing, it can effectively reduce the difference in resource utilization among clusters; in cost control, it achieves a significant reduction in task execution cost; in terms of task overdue time, it greatly shortens the average completion time of tasks, thus providing a more efficient, intelligent, and reliable solution for cloud computing task scheduling.

Comment 5: Motivation statements are not written in the manuscript.

Answer: Thanks for good advice. Please refer to section “Introduction” for relevant content.

This research proposes an innovative Hierarchical Intelligent Task Scheduling framework (HITS) based on the hierarchical deep reinforcement learning algorithm to ad-dress the challenge of large-scale task scheduling in cloud computing. Compared with traditional methods, HITS exhibits significant advantages. Firstly, through hierarchical partitioning and effective manipulation of the solution space, it accelerates the task scheduling process and simultaneously optimizes the task overdue time and cost, which is particularly crucial in large-scale task scheduling scenarios. Secondly, the model struc-ture and return function of deep reinforcement learning are meticulously designed in ac-cordance with the dynamic characteristics of the cloud environment. In response to the dynamic variation in the number of virtual machines, by modeling the Gaussian dis-tribu-tion of relevant features and using it as state information, the model can adaptively adjust. For different load conditions, a unique reward function is designed, which feeds back re-wards based on the load to drive the model to learn corresponding deci-sion-making strategies, thereby achieving efficient and intelligent task scheduling in the complex and variable cloud environment.

Comment 6: Literature review is poor and research gap is not identified properly.

Answer: Thanks for good advice. Please refer to section “Related work” for relevant content.

Although certain progress has been achieved in these studies, there still exist some gaps or limitations, especially in terms of insufficient adaptability to dynamic environ-ments and limited capabilities in handling complex resource management issues. In view of the above-mentioned challenges and limitations, this research proposes a Hierarchical Intelligent Task Scheduling framework (HITS) based on the hierarchical deep reinforce-ment learning algorithm. The complexity and dynamism of the cloud computing environment, as well as the need to efficiently handle large-scale tasks while satisfying diverse Quality of Service (QoS) constraints, form the foundation of this research. In the proposed HITS framework, a group of Virtual Machines (VMs) is defined as a VM cluster. When a task request arrives, it is first allocated to a cluster and then assigned to a specific VM through the task scheduler within the cluster. By applying the deep reinforcement learn-ing technology to the scheduler and meticulously designing the state space and return function of each layer, this framework can adapt to the dynamic changes of the cloud computing environment and continuously adjust its scheduling strategy through learning and network parameter updates.

Comment 7: Deep Reinforcement learning technique was not properly mapped with respect to scheduling process.
Answer: Thanks for good advice. Please refer to section “3.2 System Model” for relevant content.

Firstly, at each decision point of task scheduling, the scheduler, as the agent in reinforcement learning, interacts with the cloud environment. The agent acquires the state information of the cloud environment, which includes task attributes (such as resource requirements, expected completion time, etc.), cluster resource status (such as computing power, bandwidth, number of idle virtual machines, etc.), and detailed information of virtual machines (such as current load, processing speed, cost, etc.). Through the integration and processing of this multi-dimensional information, a state space is constructed that can comprehensively reflect the matching relationship between tasks and resources as well as the dynamic changes of the cloud environment.

Based on this state space, the agent outputs an action according to the policy network in the deep reinforcement learning model, and this action is the decision to allocate the task to a specific cluster or virtual machine. After this decision is executed in the cloud environment, the environment feeds back a reward signal to the agent. This reward signal is calculated based on our meticulously designed return function, which comprehensively considers multiple objectives such as task overdue time, cost, and load balancing. If a scheduling decision can reduce task overdue time, lower cost, and contribute to load balancing, the agent will receive a relatively high reward; otherwise, it will receive a lower reward or even a penalty.

By continuously repeating this interaction process between the agent and the environment, the deep reinforcement learning model utilizes the experience replay mechanism to store historical interaction data and updates the model parameters based on the temporal difference method, enabling the model to gradually learn the optimal task scheduling strategy under different cloud environment states. In this way, the deep reinforcement learning technology is deeply integrated into the entire task scheduling process, from state perception, decision-making to strategy optimization, realizing the intelligent and dynamic management of cloud task scheduling.

Comment 8: Mathematical modelling is weak in the manuscript

Answer: Thanks for good advice. Please refer to section “3.1 Deep Q-Learning Technique” for relevant content.

In the DQN algorithm, the most important part is how to solve the parameter θ. Typically, DQN models are trained using the time difference algorithm (TD), whose core is to update old estimates with partially fact based estimates (adjusting the parameter (θ). In other words, the loss function of typical deep learning algorithms is calculated based on the functional relationship between predicted data and labeled data, while DQN algorithms can incorporate historical empirical data as part of labeled data. Therefore, the first step in training is to calculate the target value based on the Bellman equation:

| $Q_{\text{t}\arg et}\text{ =}R_{t}+\lambda\max_{A_{t+1}} Q(S_{t+1},A_{t+1},\theta)$ | (1) |
| --- | --- |

Among them, St+1 represents the next state of the environment, At+1 represents the next action to be executed by the agent, Rt is the reward feedback from the environment after the current action is executed, and Qtarget represents the target value to be fitted by the neural network. The second step is to calculate the squared difference between it and the predicted value output by the neural network to define the loss function, and further solve for the parameter θ, which can be expressed as follows:

| $L(\theta)\text{ =}E\left[(R+\lambda\max_{A_{t+1}} Q(S_{t+1},A_{t+1},\theta)-Q(S_{t},A_{t},\theta))^{2}\right]$ | (2) |
| --- | --- |

Among them, Q (St, At, θ) is the output of the current neural network, denoted as Qprediction. Step three, calculate the gradient of the loss function as follows:

| $\frac{\partial L(\theta)}{\partial(\theta)}\text{ =}E\left[R+\lambda\max_{A_{t+1}} Q(S_{t+1},A_{t+1},\theta)-Q(S_{t},A_{t},\theta)\frac{\partial Q(S_{t},A_{t},\theta)}{\partial(\theta)}\right]$ | (3) |
| --- | --- |

Finally, gradient descent updates the parameter θ:

| $\theta\leftarrow\theta+\alpha\frac{\partial L(\theta)}{\partial(\theta)}\text{ }$ | (4) |
| --- | --- |

Where α is the learning rate. By following these four steps, one update of the neural network parameters is completed. The key to training the DQN algorithm lies in updating the target value with historical empirical data while performing gradient descent. This fact based estimation improves the learning performance of the network, allowing it to approach the optimal value function and complete the agent's learning.

Comment 9: Algorithm is design is not upto the mark

Answer: Thanks for good advice. Please refer to section “3.1 Deep Q-Learning Technique” for relevant content.

The traditional RL method designs a Q-table to store the updated state action value function until it converges to the optimal strategy Q* suitable for low dimensional discrete scenarios. However, in reality, most scenarios are continuous, and applying RL algorithms can easily lead to the problem of dimensionality disaster. To address this issue, the DeepMind team proposed an innovative Deep Q-network (DQN) algorithm [31] that combines the advantages of deep neural networks and reinforcement learning to tackle the challenges faced by traditional methods in high-dimensional spaces. The main idea of this algorithm is to use neural networks to approximate the optimal value function in order to solve the problem of high-dimensional state features. Usually, this network is referred to as, where are the parameters of the neural network. At the beginning, they are randomly initialized and then continuously learn based on the experience of the agent until the predicted output values of DQN can be as close as possible to Q*(S, A) for all inputs S and A. Fig 1(b) shows the neural network structure of a typical DQN algorithm, where the input of the model is the current state of the system environment, and the output is the Q value of each action in the action space. After the model outputs, the agent will select the action with the highest Q value to execute according to the learning criteria.

Comment 10: Result discussion is poor

Answer: Thanks for good advice. Please refer to section “5. Conclusions” for relevant content.

With the increasing popularity of cloud computing services, their large and dynamic load characteristics have rendered task scheduling an NP-complete problem. We propose a hierarchical framework for large-scale online task scheduling to reduce task cost and overdue time, which utilizes the hierarchical deep reinforcement learning approach. It groups virtual machines into clusters and effectively decomposes the complex task scheduling problem through hierarchical partitioning. A scheduler based on deep re-inforcement learning is employed, and its return function flexibly trades off and optimizes be-tween task overdue time and cost according to the load dynamics of the cloud environment. This enables the scheduler to automatically learn and adopt the most appropriate scheduling strategy under different load conditions, significantly enhancing the intelligence level of task scheduling.

The uniqueness of this framework lies in the meticulously constructed state space and return function. The state space effectively models the uncertainties of the cloud environment by integrating multi-dimensional information of tasks, clusters, and virtual machines (such as resource requirements, processing capabilities, cost, etc.), and applying statistical methods like averaging and standard deviation to handle the characteristics of the dynamic environment. The return function flexibly trades off and optimizes between task overdue time and cost according to the load dynamics of the cloud environment, enabling the scheduler to automatically learn and adopt the most appropriate scheduling strategy under different load conditions, significantly enhancing the intelligence level of task scheduling.

Experiments demonstrate that it skillfully balances cost and performance. In low-load situations, costs are reduced by using low-cost nodes within the Service Lev-el Agreement (SLA) range; in high-load situations, resource utilization is improved through load balancing. Compared with classical heuristic algorithms, it effectively optimizes load balancing, cost, and overdue time, achieving a 10% overall improvement. Verified by experiments, in comparison with a variety of traditional task scheduling algorithms (such as Random, Round - Robin, Min - Min, Dominant Resource Fairness, etc.), the HITS framework exhibits distinct advantages in multiple key performance indicators. In terms of load balancing, it can effectively reduce the difference in resource utilization among clusters; in cost control, it achieves a significant reduction in task execution cost; in terms of task overdue time, it greatly shortens the average completion time of tasks, thus providing a more efficient, intelligent, and reliable solution for cloud computing task scheduling.

There are still shortcomings in the method used in this article. Firstly, the continuous learning and updating of network parameters might introduce latency, which could impact real-time task scheduling efficiency. Furthermore, the framework's performance heavily depends on the quality and quantity of training data, which might be challenging to obtain and maintain in a dynamic cloud environment.
